# Polyketide synthases mutation in tuberculosis transmission revealed by whole genomic sequence, China, 2011–2019

**DOI:** 10.3389/fgene.2023.1217255

**Published:** 2024-01-08

**Authors:** Ting-Ting Wang, Yuan-Long Hu, Yi-Fan Li, Xiang-Long Kong, Ya-Meng Li, Ping-Yi Sun, Da-Xing Wang, Ying-Ying Li, Yu-Zhen Zhang, Qi-Lin Han, Xue-Han Zhu, Qi-Qi An, Li-Li Liu, Yao Liu, Huai-Chen Li

**Affiliations:** ^1^ Shandong University of Traditional Chinese Medicine, Jinan, China; ^2^ Department of Pulmonary and Critical Care Medicine, The Third Affiliated Hospital of Shandong First Medical University (Affiliated Hospital of Shandong Academy of Medical Sciences), Jinan, China; ^3^ Shandong Artificial Intelligence Institute Qilu University of Technology (Shandong Academy of Sciences), Jinan, China; ^4^ Jining Medical University, Jining, China; ^5^ People’s Hospital of Huaiyin Jinan, Jinan, China; ^6^ Shandong First Medical University and Shandong Academy of Medical Sciences, Jinan, China; ^7^ Department of Respiratory and Critical Care Medicine, Shandong Provincial Hospital Affiliated to 11 Shandong University, Shandong Provincial Hospital Affiliated to Shandong First Medical University, Jinan, Shandong, China

**Keywords:** *Mycobacterium tuberculosis*, mutation, polyketide synthases, transmission, phylogenetic analysis

## Abstract

**Introduction:** Tuberculosis (TB) is an infectious disease caused by a bacterium called *Mycobacterium tuberculosis* (*Mtb*). Previous studies have primarily focused on the transmissibility of multidrug-resistant (MDR) or extensively drug-resistant (XDR) *Mtb*. However, variations in virulence across *Mtb* lineages may also account for differences in transmissibility. In *Mtb*, polyketide synthase (PKS) genes encode large multifunctional proteins which have been shown to be major mycobacterial virulence factors. Therefore, this study aimed to identify the role of PKS mutations in TB transmission and assess its risk and characteristics.

**Methods:** Whole genome sequences (WGSs) data from 3,204 *Mtb* isolates was collected from 2011 to 2019 in China. Whole genome single nucleotide polymorphism (SNP) profiles were used for phylogenetic tree analysis. Putative transmission clusters (≤10 SNPs) were identified. To identify the role of PKS mutations in TB transmission, we compared SNPs in the PKS gene region between “clustered isolates” and “non-clustered isolates” in different lineages.

**Results:** Cluster-associated mutations in *ppsA, pks12,* and *pks13* were identified among different lineage isolates. They were statistically significant among clustered strains, indicating that they may enhance the transmissibility of *Mtb*.

**Conclusion:** Overall, this study provides new insights into the function of PKS and its localization in *M. tuberculosis*. The study found that ppsA, pks12, and pks13 may contribute to disease progression and higher transmission of certain strains. We also discussed the prospective use of mutant *ppsA*, *pks12*, and *pks13* genes as drug targets.

## 1 Introduction

Tuberculosis remains a major cause of suffering worldwide. Globally in 2020, tuberculosis was the second leading cause of death from infectious disease in humans worldwide, following COVID-19. Approximately 10 million individuals contracted tuberculosis disease, and roughly 1.5 million lost their lives. (Global tuberculosis report 2021, 2021). Successful TB transmission depends on the interplay of human behavior, host immune responses, and *Mycobacterium tuberculosis (Mtb)* virulence factors. More attention has been paid to the transmission of multidrug-resistant (MDR) or extensively drug-resistant (XDR) *Mtb*, (Clark et al., 2013; Yang et al., 2017a; Madikay et al., 2017; Bouzouita I Fau - Cabibbe et al., 2019; Dixit et al., 2019; Jiang et al., 2020a), or described the dynamics of TB transmission combined with host risk factors (Genestet C Fau - Tatai et al., 2019; Liu et al., 2021). To date, there has been no systematic study to delineate the role of virulence factors in TB transmission. (Global tuberculosis report 2021, 2021). In *Mtb*, polyketide synthase (PKS) genes encode large multifunctional proteins that contain all domains required to catalyze the various steps involved in the biosynthesis of complex mycobacterial lipids. These lipids have been shown to be key players for mycobacterial pathogenicity and transmissibility (Camacho et al., 1999; Cox et al., 1999; Asselineau et al., 2002; Reed et al., 2004; Tsenova et al., 2005; Astarie-Dequeker et al., 2009; Verschoor et al., 2012; Cambier et al., 2014; Passemar et al., 2014) and contributors to the cell envelope permeability barrier to antimicrobial drugs (Camacho et al., 2001; Alibaud et al., 2011; Chavadi et al., 2011; Yu et al., 2012).

**FIGURE 1:**
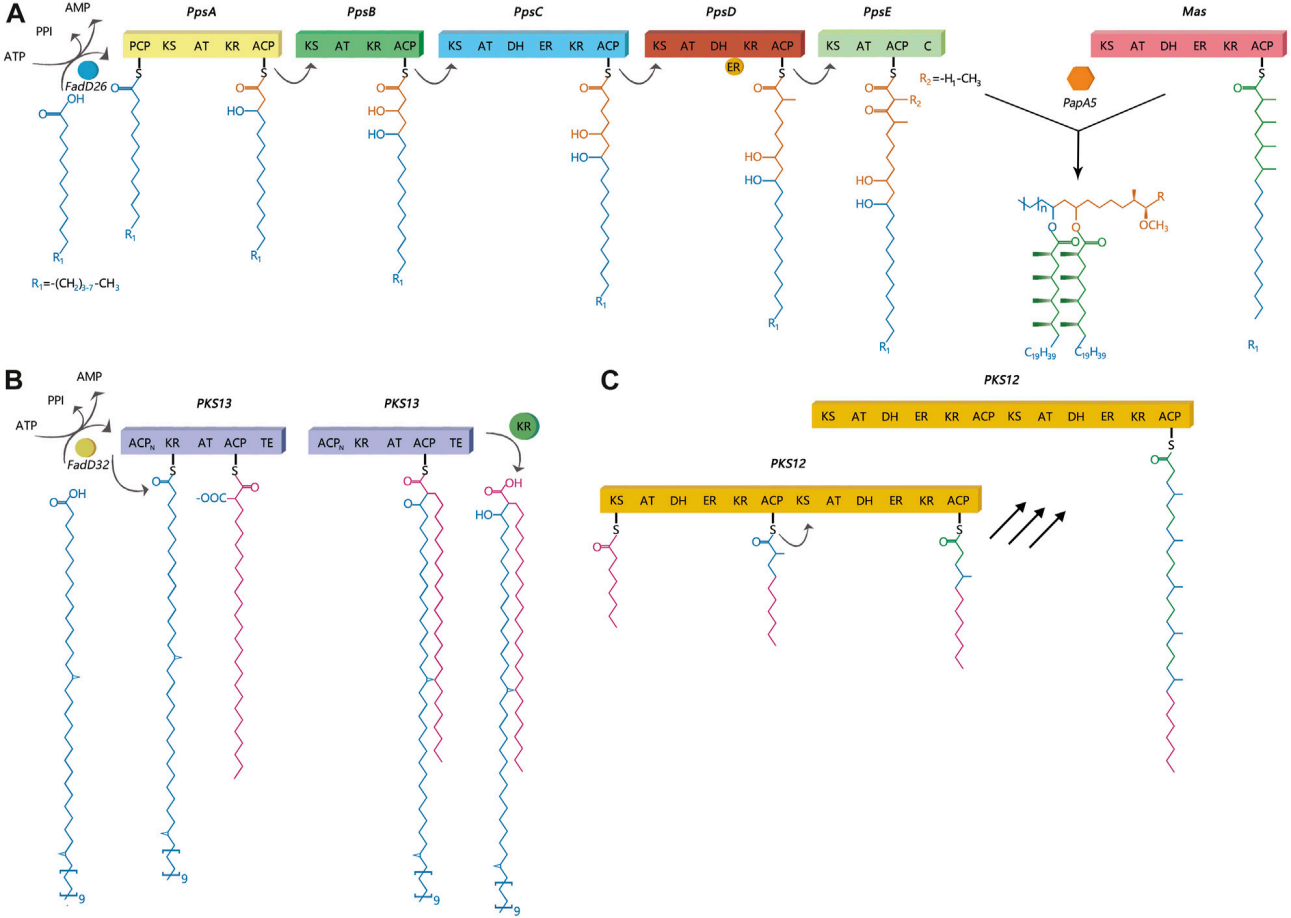
Catalytic and mechanistic versatility of ppsA, pks12, pks13. **(A)** PpsA initiates biosynthesis of phthiocerol products. It does this by extending its substrate using a malonyl-CoA extender unit; the same has been observed for ppsB and ppsC proteins. The ppsD and ppsE proteins add two (R)-methylmalonyl units to the substrate. **(B)** The pks13 protein consists of five domains, including two acyl carrier protein domains, a β-ketoacyl-synthase, an acyltransferase, and a C-terminal thioesterase (TE) domain, which together contain all the activities required for the condensation of two long-chain fatty acids. **(C)** There are two complete sets of modules in pks12, which produce mycoketide using five alternating condensations of methylmalonyl and malonyl units. The iterative process would generate a fully saturated chain with branching at each alternate ketide unit.

Polyketide synthases are grouped into three protein structure-based types: Type I, Type II, and Type III. According to a previous study, Type I PKS generally synthesizes complex metabolites with the use of a modular or iterative biosynthetic mechanism (Gokhale et al., 2007a). In an iterative mechanism, the final product is produced by repeating the same active sites, while modular proteins follow an assembly-line mechanism (Gokhale et al., 2007a). This study primarily focused on three lipids: DIMs, MPMs, and mycolic acids and their corresponding synthesis proteins, ppsA, pks12, and pks13, respectively. PpsA, pks13 and pks12 were belong to Type I PKS. PpsA and pks13 belong to modular I PKS, while pks12 belongs to iterative I PKS (Onwueme et al., 2005). Dimycocerosates are a family of compounds that contain two diols, phthiocerol and phenolphthiocerol, which have been proven to be major mycobacterial virulence factors with complex molecular mechanisms of action (Camacho et al., 1999; Cox et al., 1999; Reed et al., 2004; Tsenova et al., 2005; Astarie-Dequeker et al., 2009; Cambier et al., 2014; Passemar et al., 2014). The clusters of *ppsABCDE* genes had been shown to be involved in the biosynthesis of phthiocerol products (Figure 1A). Phthiocerol products are synthesized by catalyzing a stepwise chain elongation and functional group modification with modular organization of pps proteins (Trivedi et al., 2005; Siméone et al., 2010). As shown in Figure 1C, the pks12 protein is involved in biosynthesis of a phospholipid MPM (Matsunaga et al., 2004). Recently, it has been discovered that novel phospholipid MPMs isolated from *Mtb* and other pathogenic mycobacteria consist of a mannosyl-β-1-phosphate. Mycolic acids are key players in the infectious process (Moody DB et al., 2002; Geisel RE et al., 2005; Layre et al., 2009; Esin et al., 2013). In mycolic acid synthesis, pks13 performs Claisen condensation of a C26 α-alkyl branch and C40–60 meromycolate precursors as the final assembly stage (Figure 1B) (Portevin et al., 2004). It has been demonstrated that this activity is crucial both *in vitro* and *in vivo* (Portevin et al., 2004; Wilson et al., 2013). Additionally, according to several genomic investigations, some PKS disruption mutants in mycobacteria have altered lipid profiles and some also show virulence attenuation (Sirakova et al., 2001; Dubey et al., 2002). PKS proteins play a significant role in enhancing the virulence and pathogenicity of *M.tb*. Nonetheless, the exact regulatory mechanism of PKS in *M.tb* is still unclear, and there is limited research on how gene mutations affecting PKS impact the transmission of *M.tb*. Thus, to develop effective TB control strategies, it is also necessary to gain a deeper understanding of the role of PKS gene in TB transmission. Therefore, this study aimed to identify the role of PKS mutations in TB transmission and assess its risk and characteristics. We also discussed the prospective use of mutant PKS genes as drug targets.

## 2 Materials and methods

### 2.1 Clinical isolates

**FIGURE 2:**
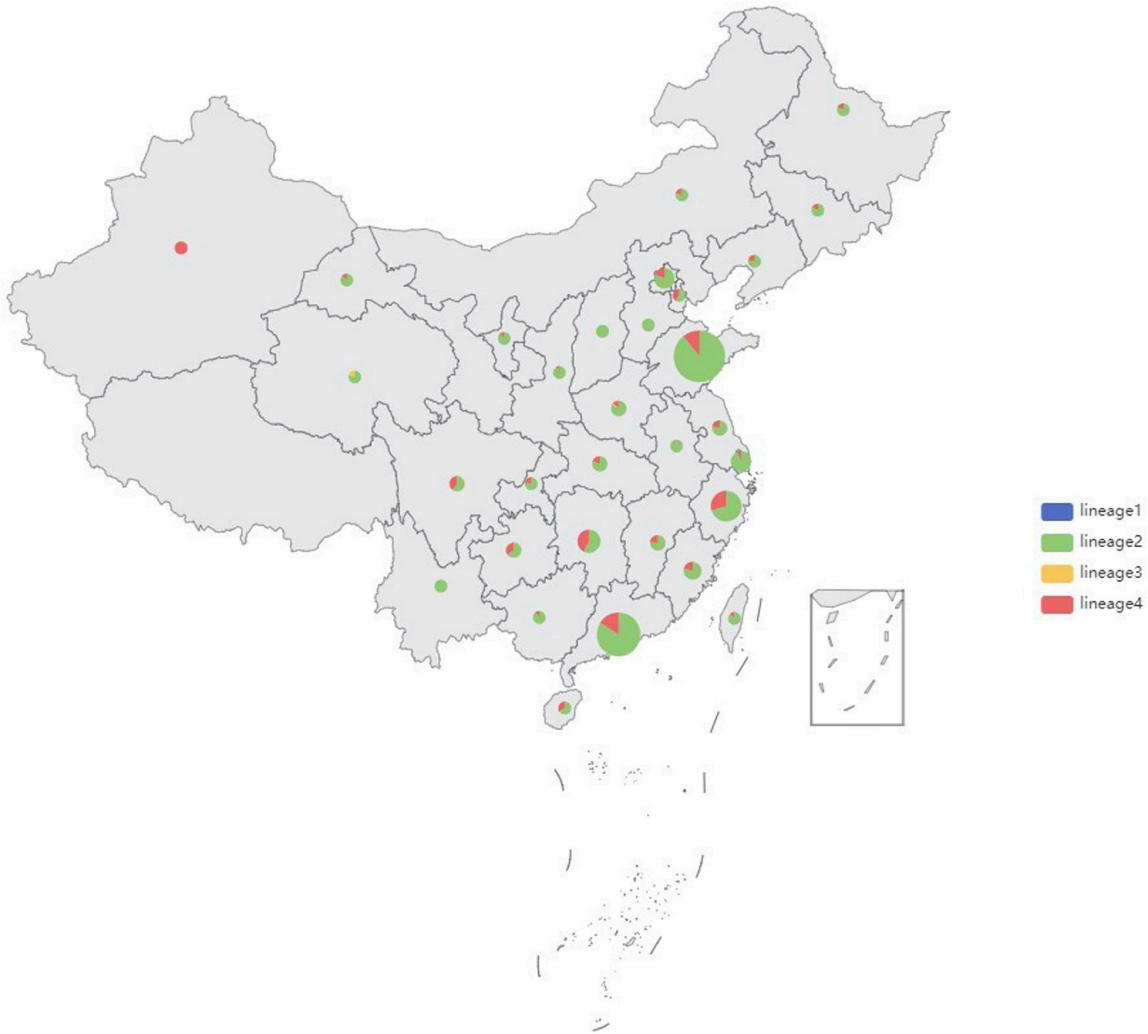
Sample size and lineages proportion in different regions of the 3,204 isolates, China, 2011–2019.

**FIGURE 3:**
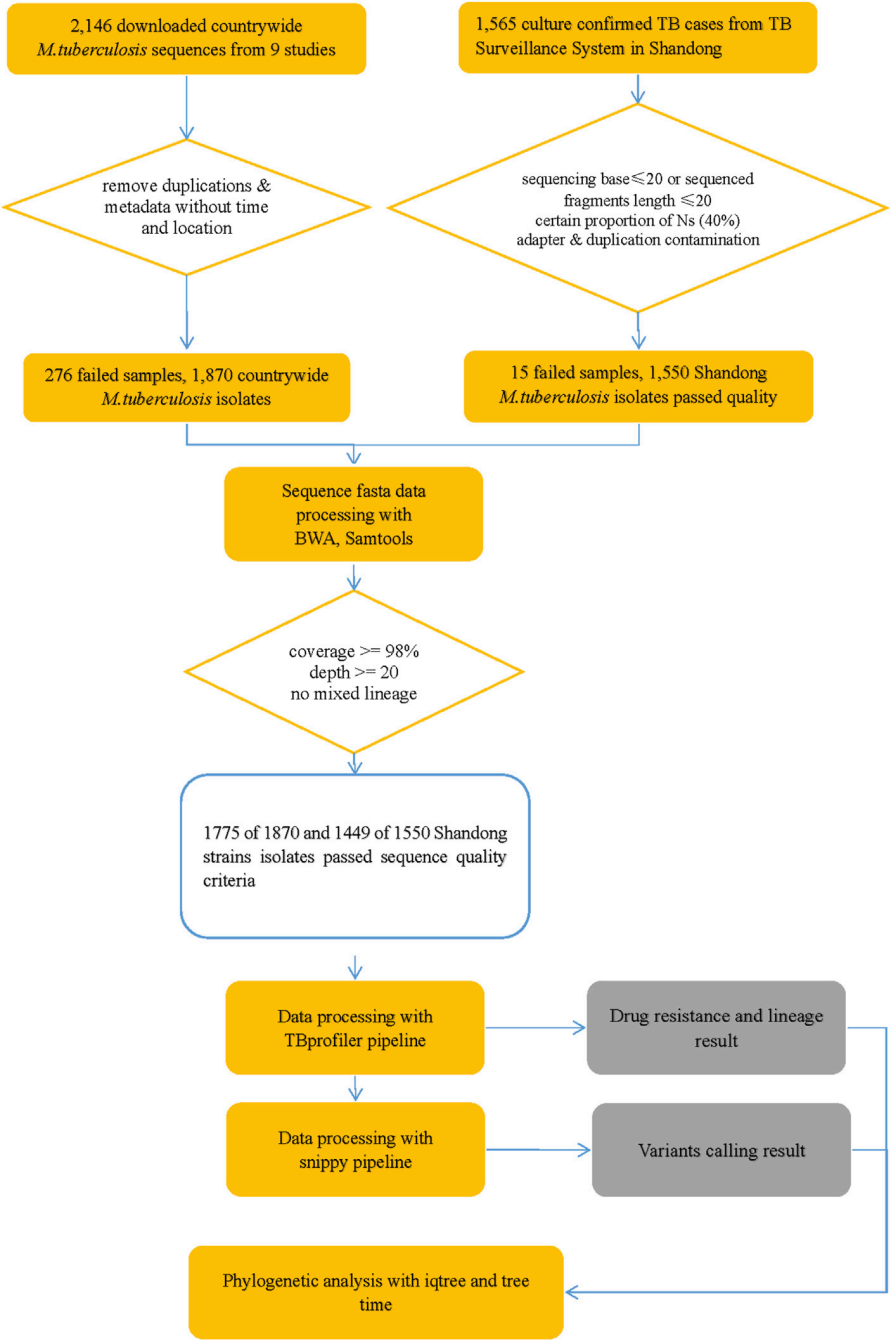
Flowchart 1: a flowchart about the process of identification and exclusion of genomic data. *M tuberculosis*, *Mycobacterium tuberculosis*; TB, tuberculosis.

Genomic DNA was successfully extracted from 1,468 *Mtb* samples from Shandong Provincial over a 5-year period for this study, and a total of 1,449 samples passed quality control (QC). Quality control of sequenced reads was carried out using FastQC software. In this study, we combined the 1,449 *Mtb* whole genome dataset with another genome dataset consisting of 1755 isolates, which were acquired from nine previously published articles (Zhang et al., 2013; Luo et al., 2015; Yang et al., 2017b; Liu et al., 2018a; Hicks et al., 2018; Yang et al., 2018; Chen et al., 2019; Huang et al., 2019; Jiang et al., 2020b). These samples were randomly collected from 21 provinces, 4 municipalities, and 5 autonomous regions in China, totaling to 3,204 isolates, from 2011 to 2019, to analyze the role of PKS mutation in TB transmission. Of the 3,204 *Mtb* isolates, Shandong contributed the most isolates (1,484), Yunnan the fewest (2), Xinjiang and Hainan (3), Qinghai and Tianjin (5), Gansu (8), Chongqing (9) and other provinces, municipalities, or autonomous regions contributed from 11 to 454 isolates; 73 had undetermined sources (Figure 2). We added a Supplementary Table S1) of the list of the1755 isolates, together with their corresponding meta-data. We also added a flowchart (Figure 3) about the process of identification and exclusion of genomic data.

### 2.2 Whole-genome sequencing and SNP identification

The genome was sequenced using HiSeq 4,000 (Illuminia Inc., San Diego, CA, United States). We discarded low-quality raw reads from paired-end sequencing. Maximal Exact Match algorithm was implemented by bwa mem (version 0.7.17-r1188) and was used to align the read to the H37Rv reference genome (NC_000962). Samclip (version 0.4.0) and samtools markdup (version 1.15) were used to remove clamped alignments and duplicated reads, excluding samples with coverage less than 98% and depth less than 20 (Li and Durbin, 2009; Li et al., 2009; Li and Durbin, 2010; Li, 2013). Variant calling was performed using Freebayes (version 1.3.2) and bcftools (version 1.15.1) with a filter parameter ‘FMT/GT = “1/1”&& QUAL>=100 && FMT/DP>=10 && (FMT/AO)/(FMT/DP)>=0’. Single nucleotide polymorphisms in previously defined repetitive regions were excluded, including *PPE* and *PE-PGRS* genes, and mobile elements or repeat regions and repeat bases generated by TRF (version 4.09) and Repeatmask (version 4.1.2-p1) (Benson, 1999; Saha et al., 2008; Garrison and Marth, 2012; Danecek et al., 2021). The filtered vcf file was annotated using snpEff (version 4.3t) to get the final SNP samples (http://SnpEff.sourceforge.net/) (Cingolani et al., 2012). Genotypic drug resistance of each isolate was predicted in TBProfiler using an established library of mutations (https://github.com/jodyphelan/tbdb) (Coll et al., 2015). The virulence factor database (http://www.mgc.ac.cn/VFs/) contains various medically important bacterial pathogen virulence factors, which include 86 experimentally confirmed and 171 putative genes related to the virulence of *Mtb* (Liu et al., 2022). There are at least 24 different PKS encoded in the genome (Cole et al., 1998).

### 2.3 *Mtb* lineage and genomic cluster

We used the web-based tool TBProfiler (version 4.3.0) to analyze 3204 *M. tuberculosis* WGS data to assign lineages and predict drug resistance (Phelan JE et al., 2019). Genomic clusters were ascertained independently of the epidemiological data, and Genomic clusters were inferred based on how genetically similar two isolates were from each other. The upper thresholds of genomic relatedness or cluster is defined as 12 SNPs or alleles cut off or less and a recent transmission event is defined as 5 or less SNPs or alleles (Walker et al., 2013; Kohl TA et al., 2018). If two isolates exhibited a distance of more than 12 SNPs or alleles, they were called unique strains. In this study *M. tuberculosis* isolates with a genomic difference (s) ≤ 10 single nucleotide polymorphisms (SNPs) were defined as a genomic cluster (Yang et al., 2017a) for further analysis of transmission cluster to avoid missing cases and incorporating recent and old transmission events, which is similar to definitions used in previous genomic studies of *M. tuberculosis* transmission (Walker et al., 2013; Walker et al., 2014; Guerra-Assunção et al., 2015). As suggested by recent analysis of intra-patient variation, the estimate of 5 SNPs may be too low (Lieberman TD et al., 2016), we finally chose the cut-off of 10 SNPs to define transmission clusters for further analysis based on the previous study (Holt et al., 2018). The clustering was performed based on the statistical analysis which was not associated with sampling.

### 2.4 Phylogenetic analysis

Reference genome with only substitution variants instantiated was used as the sample’s genome. Maximum-likelihood (ML) phylogenetic trees were constructed and dated by IQ-TREE (v1.6.12) model “JC + I + G4” with 1,000 ultrafast bootstrap replicates and treetime (v0.9.0) [GitHub - neherlab/treetime: Maximum likelihood inference of time stamped phylogenies and ancestral reconstruction. https://github.com/neherlab/treetime.] (Zelner et al., 2016)The trees were constructed using the highest likelihood model selected by automatic model selection in IQ-TREE (v1.6.12), which utilized the JC model of nucleotide substitution and invariable site plus discrete Gamma model of rate heterogeneity to analyze the genome samples with only substitution variants replaced in reference sequence. Sampling dates were used to construct a temporal phylogeny using TreeTime (v0.9.0) [GitHub - neherlab/treetime: Maximum likelihood inference of time stamped phylogenies and ancestral reconstruction. https://github.com/neherlab/treetime.] (Zelner et al., 2016), and tip-randomization was performed to confirm the presence of a strong temporal signal. Bayesian evolutionary analyses were conducted to identify the best substitution, clock, and demographic models, with marginal likelihood estimates used for model selection. The visualization of the bacteriological information was performed using Interactive Tree of Life (Version 6.6) (Letunic and Bork, 2021).

### 2.5 Statistical analysis

The mutation loci in the polyketide synthesis gene region between “clustered isolates” and “non-clustered isolates” was compared using univariate and multivariate logistic regression analysis in different lineages. Factors with a *p*-value less than 0.05 in the final model were considered to be independently associated with genomic clusters. The odds ratios (OR) and 95% confidence intervals (95% CI) were calculated. All statistical analyses were performed in R version 4.2.0 unless otherwise stated. Finally, a sensitivity analysis was performed to determine whether there was a rank correlation between cluster size and clustering rate with ordered logistic regression analysis. The R code see Supplementary Materials 2. Only fixed mutations (25%≤frequency<100%) were calculated from different lineages. The mutation frequency was calculated as the percentage of mutation isolates among the number of total isolates in different lineages. The detailed mutations were indicated in Table 3. The clustering rate was calculated as the percentage of cluster isolates among total isolates (number of cluster isolates/number of total isolates). Only nonsynonymous mutations were analyzed. Insertions and deletions were excluded from the analysis as they are often the result of errors in genome assembly. In terms of SNPs, isolates that possess the mutation in the PKS gene region are referred to as mutation isolates.

### 2.6 Predicted impact of mutations on proteins

Protein prediction algorithm, I-Mutant v2.0 (http://folding.biofold.org/i-mutant/i-mutant2.0.html), was used to predict the functional impact of noteworthy SNPs on protein structure and function.

### 2.7 Genomic data availability

The newly sequenced whole genome dataset of 1,449 *M. tuberculosis* strains was deposited in the NCBI Bio Project (https://www.ncbi.nlm.nih.gov/sra/), and 1755 other isolates were downloaded from the European Nucleotide Archive repository (Supplementary Table S1). Additional data can be obtained by contacting the corresponding authors upon request.

## 3 Results

### 3.1 Genetic diversity

**FIGURE 4:**
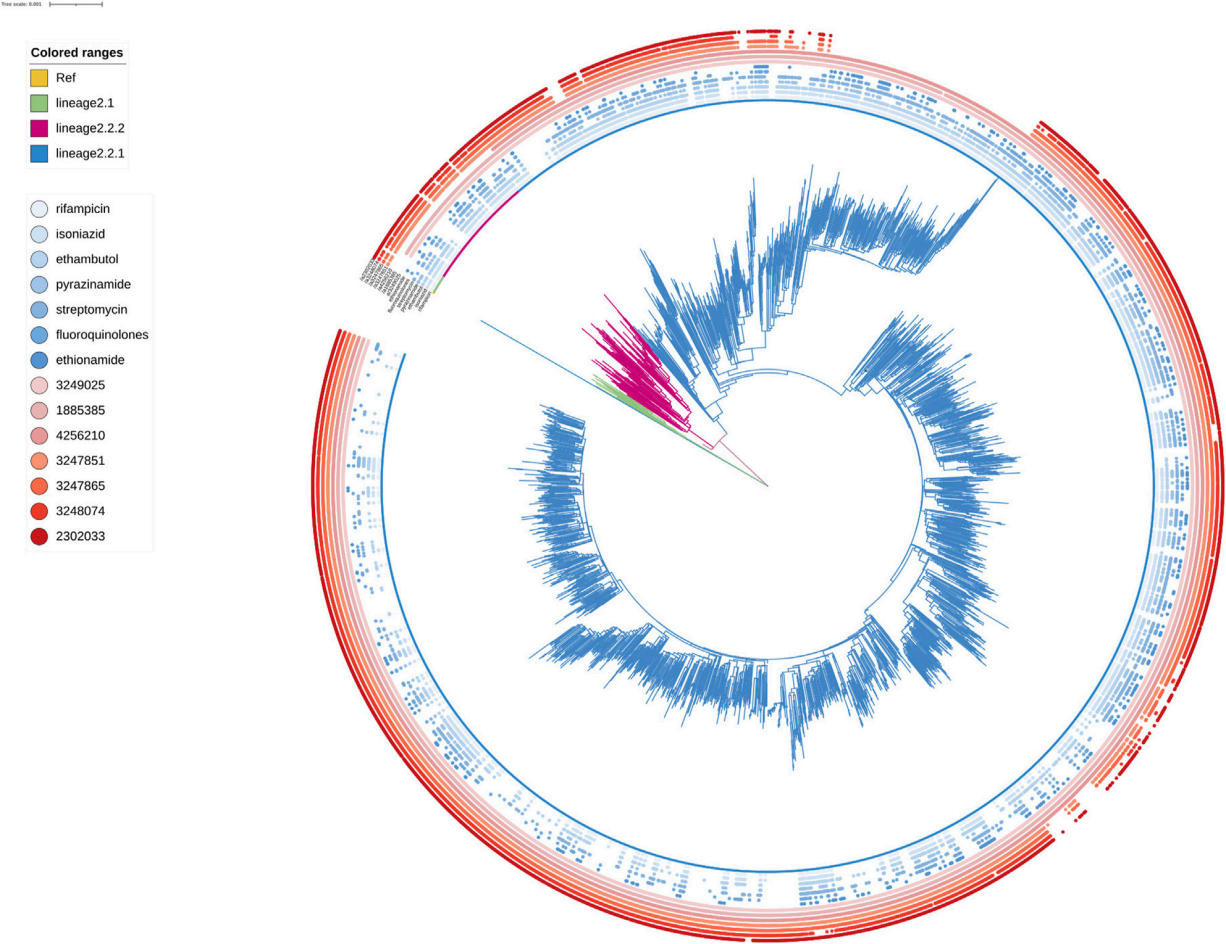
Phylogenetic tree for lineage2. Green, red and blue branches indicated L2.1, L2.2.2 and L2.2.1 strains, respectively. The inner blue dots indicated the resistance to known antimicrobial drugs. The outermost red dots showed the strains contained SMs.

**FIGURE 5:**
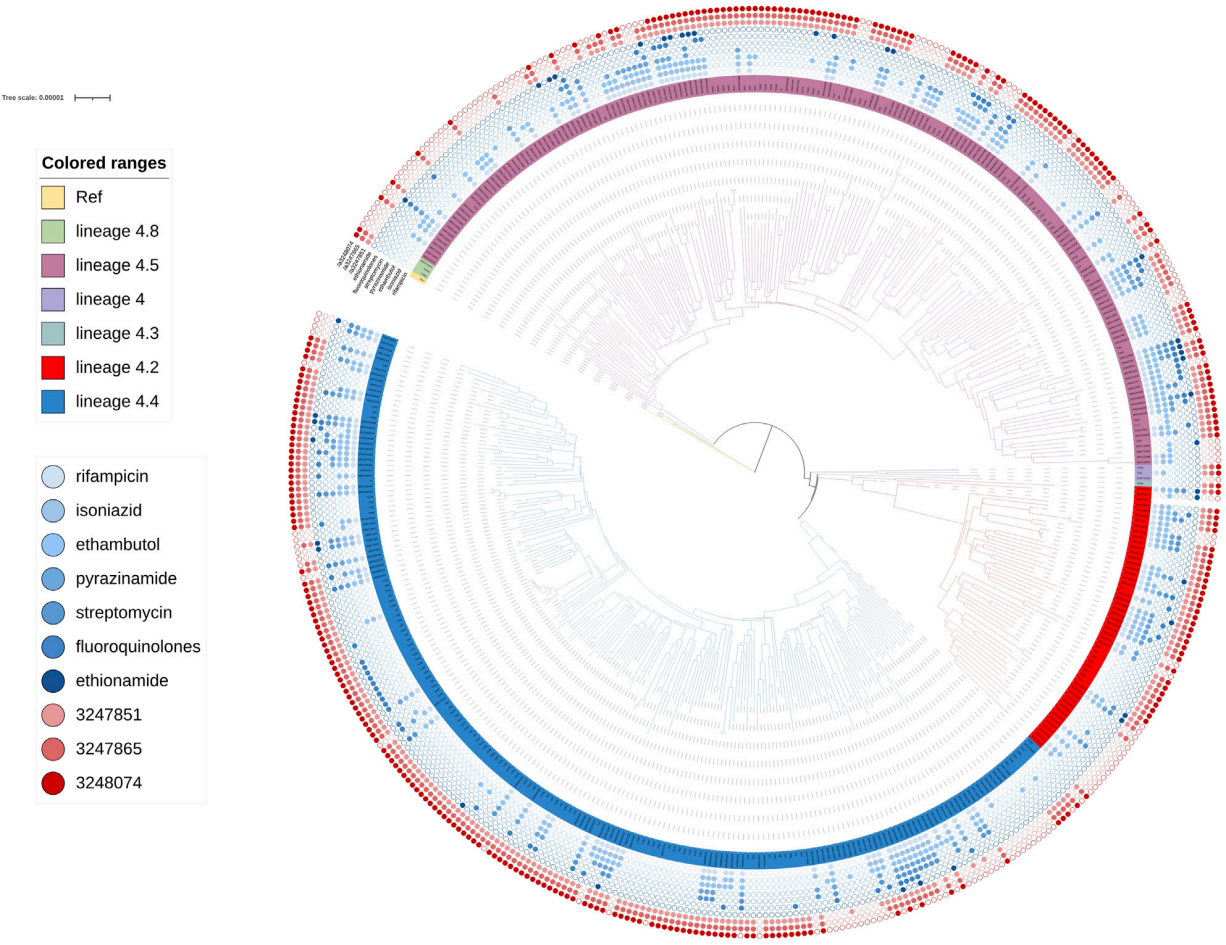
Phylogenetic tree for lineage4. Green, rose red, purple, dark green, red and blue branches indicated sublineage 4.8, sublineage 4.5, sublineage 4, sublineage 4.3 sublineage 4.2 and sublineage 4.4 strains, respectively. The inner blue dots indicated the resistance to known antimicrobial drugs. The outermost red dots showed the strains contained SMs.

shown in the map (Figure 2), 85.73% (2,745/3,204) strains belonged to Lineage 2 (Beijing lineage), 13.84% (443/3,204) to Lineage 4 (Euro-American lineage), while only 0.31% (11/3,204) to Lineage 3 (East African-Indian lineage) and 0.12% (5/3,204) to Lineage 1(Indo-Oceanic lineage). A maximum likelihood phylogenetic tree was constructed for lineage 2 and lineage 4 *Mtb* isolates (Figure 4; Figure 5).

### 3.2 Clustering rate of the *Mtb* isolates

One thousand four hundred and sixty-four out of 2,745 isolates in lineage 2 were grouped into 446 genomic clusters (Table 1). The clustering rate was 53.33%, which indicated the transmission of lineage 2 in China from 2011 to 2019. The genomic clusters consisted of 2–109 isolates. Majority of the clusters had two isolates, accounting for 36.47% (534/1,464). There were 52 genomic clusters consisting of two to nine isolates in lineage 4. The clustering rate of lineage 4 was 29.86%.

**TABLE 1:** The cluster size and the number of genomic clusters of the *Mycobacterium tuberculosis* isolates in lineage2 and lineage4.

No. of isolates in clusters	No. of clusters	No. of isolates	Proportion (%)
*Lineage2*			
0	0	1281	46.67
2	267	534	19.45
3 to 6	157	585	21.31
≥7	22	345	12.57
Total	446	2745	100
*Lineage4*			
0	0	310	70.14
2	35	70	15.84
3 to 6	16	53	11.99
≥7	1	9	2.04
Total	52	442	100

### 3.3 Drug resistance associated with genomic clusters

Known antimicrobial resistance mutations were detected in lineage 2 and lineage 4 (Table 2). Mutations in lineage 2 associated with resistance to rifampicin, isoniazid, pyrazinamide, streptomycin, ethambutol, fluoroquinolones, and ethionamide were all associated with genomic clusters (*p* < 0.05). This was the same as lineage 4, which was associated with resistance to streptomycin, isoniazid, rifampicin, and pyrazinamide and had a higher risk of clustering (*p* < 0.05). The phylogenetic trees show the drug resistance profile for 7 anti-TB drugs based on the presence of validated resistance-conferring mutations (Figure 4; Figure 5). Mutations occurred mainly in drug resistance genes such as *katG, rpoB, rpsL, embB, pncA, gyrA*, and *ethA*. Drug resistance is an important factor of TB transmission. In our study, we just used the Drug resistance mutations as exposure factors in multivariate logistic regression analysis to improve the sensitivity of analysis results.

**TABLE 2:** Known antimicrobial resistance mutations associated with genomic clusters of lineage 2 and lineage 4.

Antimicrobial	No. of isolates	Clustering percetenge (%)	OR	P	Mutation genes
*Lineage2*					
rifampicin	1,203	43.83	1.897 (1.626, 2.211)	**<0.001**	*rpoB, rpoC*
isoniazid	1,264	46.05	1.897 (1.626, 2.211)	**<0.001**	*katG, fabG1, ahpC, inhA*
pyrazinamide	519	18.91	1.552 (1.276, 1.887)	**<0.001**	*pncA*
streptomycin	1,021	37.19	1.753 (1.497, 2.053)	**<0.001**	*rpsL, rrs, gid*
ethambutol	744	27.10	1.746 (1.500, 2.033)	**<0.001**	*embB, embA*
fluoroquinolones	458	16.68	1.565 (1.274, 1.924)	**<0.001**	*gyrA, gyrB*
ethionamide	363	13.22	1.267 (1.013, 1.585)	**0.038**	*fabG1, ethA, inhA*
*Lineage4*					
rifampicin	180	40.72	1.719 (1.139, 2.596)	**0.01**	*rpoB, rpoC*
isoniazid	180	40.72	1.719 (1.139, 2.596)	**0.01**	*katG, fabG1, ahpC*
pyrazinamide	109	24.66	1.786 (1.134, 2.814)	**0.012**	*pncA*
streptomycin	70	15.84	1.847 (1.091, 3.129)	**0.022**	*rpsL, rrs, gid*
ethambutol	189	42.76	1.333 (0.885, 2.009)	0.169	*embB, embA*
fluoroquinolones	75	16.97	1.507 (0.896, 2.534)	0.122	*gyrA, gyrB*
ethionamide	38	8.60	0.826 (0.389, 1.752)	0.618	*fabG1, ethA, inhA*

OR, odds ratio. The bold values mean these mutations were statistically significant.

### 3.4 Spread mutation (SM)

As shown in Table 3, the univariate logistic analysis detected eight loci mutations in the PKS gene region of L2 isolates, which were statistically significant (*p* < 0.05). Seven were risk factors (OR>1) and one was a protective factor (OR<1). The seven risk factors [ppsA(3,248,074, 3,247,851, 3,247,865, 3,249,025), pks12(2,302,033), pks13(4,256,210) and pks8(1,885,385)] were defined as Spread Mutations (SMs), meaning isolates with the seven SMs were more likely to be clustered than those without. The basic information was shown in Table 4. All seven SMs were found in L2 and three SMs were found in L4 [ppsA(3248074,3247865,3,247,851)].

**TABLE 3:** Univariate regression analysis on SMs associated with clustering in PKS gene region of lineage 2 and lineage 4^a^

	*Lineage2*					*Lineage4*				
Genomic position	No. of isolates	Mutation frequency	Clustering rate *	OR	P	No. of isolates	Mutation frequency	Clustering rate *	OR	P
*ppsA*										
3,248,074	2025	73.77%	54.56%	1.21 (1.02,1.43)	**0.03**	282	63.80%	33.33%	1.61 (1.04, 2.51)	**0.035**
3,247,851	2,298	83.71%	55.48%	1.70 (1.39,2.09)	**<0.001**	315	71.27%	33.02%	1.74 (1.09, 2.86)	**0.024**
3,247,865	2,279	83.02%	55.59%	1.71 (1.40,2.09)	**<0.001**	310	70.14%	33.55%	1.88 (1.17, 3.07)	**0.01**
3,249,025	2,723	99.19%	53.65%	7.33 (2.49,31.3)	**0.001**	0	0	0	0	0
3,247,316	2,733	99.56%	53.24%	0.38 (0.08,1.28)	0.15	440	99.55%	29.77%	0.42 (0.02, 10.80)	0.55
*Pks12*										
2,302,033	2,234	81.38%	55.73%	1.68 (1.38,2.04)	**<0.001**	22	5%	40.91%	1.67 (0.70,4.01)	0.25
2,296,042	2,730	99.45%	53.26%	0.57 (0.18,1.61)	0.31	250	56.56%	28.00%	0.82 (0.54, 1.23)	0.33
2,300,546	2,607	94.97%	52.89%	0.70 (0.49,0.99)	**0.047**	399	90.27%	28.82%	0.62 (0.33, 1.20)	0.15
2,296,297	0	0	0	0	0	112	25.34%	26.79%	0.82 (0.50, 1.31)	0.41
*Pks13*										
4,256,210	2,582	94.06%	54.11%	1.69 (1.23,2.34)	**0.001**	0	0	0	0	0
4,258,106	2,209	80.47	53.87%	1.12 (0.92,1.35)	0.25	0	0	0	0	0
*Pks6*										
485,810	2,738	99.75	53.25%	0.19 (0.01,1.11)	0.12	0	0	0	0	0
488,579	0	0	0	0	0	196	44.34%	29.59%	1.04 (0.69, 1.57)	0.84
*Pks7*										
1,877,744	2,744	99.96%	53.35%	#	0.95	0	0	0	0	0
1,881,343	0	0	0	0	0	185	41.86%	31.35%	1.13 (0.75, 1.70)	0.56
*Pks8*										
1,885,772	2,739	99.78%	53.27%	0.23 (0.01,1.42)	0.18	440	99.54%	30.00%	#	0.98
1,885,385	2,718	99.02%	53.57%	2.74 (1.24,6.66)	**0.017**	0	0	0	0	0
*Pks15*										
3,296,371	1803	65.68%	52.52%	0.91 (0.78,1.07)	0.24	0	0	0	0	0
3,296,843	2,728	99.38%	53.19%	0.35 (0.10,0.99)	0.066	440	99.55%	29.77%	0.42 (0.02, 10.80)	0.55
*ppsD*										
3,267,163	0	0	0	0	0	183	41.40%	31.69%	1.16 (0.77, 1.75)	0.48
*Pks1*										
3,295,663	0	0	0	0	0	127	28.73%	30.71%	1.06 (0.67, 1.65)	0.81
Pks3										
1,315,191	2,745	100%	53.33%	#	#	442	100%	29.86%	#	#
*Pks5*										
1,722,228	2,725	99.27%	53.36%	1.14 (0.47,2.80)	0.76	0	0	0	0	0

^a^ sMs refer to seven loci mutations statistically significant (*p* < 0.05) which are risk factors in the PKS, gene region of lineage2 isolates. *Genomic position are genomic nucleotide positions in Mtb H37Rv genome NC_000962. * The clustering rate was calculated as the percentage of cluster isolates among total isolates (number of cluster isolates/number of total isolates). #means there is no result in statistical software or the result was too large and nonsense. OR, odds ratio. The bold values mean these mutations were statistically significant.

**TABLE 4:** The basic information of the SMs associated with clustering in PKS gene of lineage 2 and lineage 4.

Genomic position	Type	References	Variant	Gene
*Lineage2*				
3,248,074	mnp	GC	AT	ppsA
3,247,851	complex	GCCCGG	ACTCGC	ppsA
3,247,865	complex	GCAAA	TAGGG	ppsA
3,249,025	snp	T	G	ppsA
2,302,033	snp	G	A	pks12
4,256,210	snp	G	T	pks13
1,885,385	snp	T	G	pks8
*Lineage4*				
3,248,074	complex	GC	AT	ppsA
3,247,851	complex	GCCCGG	ACTCGC	ppsA
3,247,865	complex	GCAAA	TAGGG	ppsA

The clustering rate of lineage 2 was 53.33%, while lineage 4 was 29.86%. Lineage 2 exhibited a higher clustering rate than lineage 4 (Table 1), which was determined that the isolates of L2 spread faster than those of L4. The SNPs of lineage 2 and lineage 4 were not exactly the same. Some SNPs were found in lineage 2 but not found in lineage 4. The vast majority of these SNPs of lineage 2 exhibit high clustering rate (above 52.52%). Similarly, some SNPs were found in lineage 4 and not found in lineage 2. The clustering rate of these SNPs of lineage 4 ranged from 26.79% to 40.91%. We found seven SMs in lineage 2, while three SMs in lineage 4. However, owing to the smaller sample size of L4, we cannot guarantee that there were no hidden SMs. Interestingly, the clustering rate of SNP [pks12(2302033)] was higher than that of other SNP in lineage 4, but it was not statistically significant (*p* < 0.05) in univariate logistic analysis. We think it was because the amount of mutation isolates that contained SNP[pks12(2302033)] was too small.

We found four SMs in lineage 2 were statistically significant, while none in lineage 4 in multivariable regression analysis (Table 5). Due to the large standard error, P and OR were undetermined, and this can be due to the small sample size of lineage 4. In multivariable regression analysis, factors independently associated with genomic clusters including SMs and antimicrobial resistance mutations associated with genomic clusters of different lineages were introduced into the statistical model. *PpsA* (3249025), *pks12* (2302033) and *pks13* (4256210) are risk factors, while *ppsA* (3248074) was protect factor. Notably, the OR of *ppsA* (3249025) in lineage 2 were larger and the mutation was more likely to be clustered compared to other SMs.

**TABLE 5:** Multivariable regression analysis on SMs associated with clustering in PKS gene region of lineage 2 and lineage 4.

Genomic position	P	Or (95%CI)
*Lineage2*		
3,249,025	**0.005**	37.743 (3.060, 465.584)
2,302,033	**<0.001**	2.251 (1.487, 3.408)
4,256,210	**0.006**	1.643 (1.154, 2.340)
3,247,865	0.642	1.229 (0.515, 2.934)
3,248,074	**0.042**	0.742 (0.557, 0.989)
3,247,851	0.907	0.951 (0.411, 2.201)
1,885,385	0.07	0.133 (0.015, 1.179)
rifampicin	**0.001**	1.749 (1.271, 2.407)
pyrazinamide	0.462	0.904 (0.691, 1.183)
streptomycin	0.074	1.234 (0.980, 1.554)
fluoroquinolones	0.144	1.202 (0.939, 1.539)
ethambutol	0.752	0.957 (0.729, 1.257)
isoniazid	0.514	1.109 (0.813, 1.514)
ethionamide	0.176	0.834 (0.640, 1.085)
*Lineage4*		
3,247,865	0.999	a
3,248,074	0.832	1.096 (0.471, 2.550)
3,247,851	0.999	a
rifampicin	0.313	1.342 (0.758, 2.377)
pyrazinamide	0.591	1.211 (0.602, 2.436)
streptomycin	0.506	1.272 (0.626, 2.586)

^a^ Means there is no result in statistical software or the result was too large and nonsense. OR, odds ratio. The bold values mean these mutations were statistically significant.

Our study attempts to identify mutations that increase transmissibility. Lineage 2.2.1(Beijing lineage) strains are more transmissible than other *Mtb* lineages (Holt et al., 2018). Genomic evidence for enhanced transmission of the Beijing lineage has been documented in Russia (associated with antimicrobial resistance) (Casali et al., 2014) and Malawi (independent of antimicrobial resistance) (Guerra-Assunção JA et al., 2015). We also analyzed the SMs in lineage 2.2.1strains (Table 6). There are four SMs in lineage 2.2.1 strains. Only one SM [*pks12* (2302033)] was statistically significant (*p* < 0.05) in multivariable regression analysis. And the data showed that the SMs in lineage 2.2.1 have higher clustering rate than other lineages which are predicted to be more transmissible.

**TABLE 6:** Ordinal regression analysis on SMs associated with clustering in PKS gene region.

Genomic position*	Value	Std.Error	T value	Ordered analysis	
				Or (95% CI)	P
*Lineage 2*					
*ppsA*					
3,249,025	3.5	0.91	3.86	33.069 (5.914,220.171)	**<0.001**
3,248,074	−0.41	0.12	−3.39	0.665 (0.525,0.842)	<0.001
3,247,865	0.41	0.39	1.04	1.505 (0.703,3.329)	0.15
3,247,851	−0.08	0.38	−0.22	0.92 (0.422,1.929)	0.415
*Pks12*					
2,302,033	0.68	0.19	3.49	1.973 (1.351,2.901)	**<0.001**
*Pks13*					
4,256,210	0.33	0.17	1.97	1.389 (1.005,1.934)	**0.024**
*Pks8*					
1,885,385	−1.71	0.64	−2.68	0.181 (0.051,0.661)	**<0.001**
*Lineage 4*					
*ppsA*					
3,248,074	0.15	0.39	0.38	a	a
3,247,865	7.92	35.38	0.22	a	a
3,247,851	−7.48	35.38	−0.21	a	a

^a^ The standard error of regression coefficient in Lineage4 was too large. The bold values mean these mutations were statistically significant.

Evolutionary convergence has previously been used as a signal of positive selection to identify mutations associated with antimicrobial resistance in Mtb (Hazbón et al., 2008; Farhat MR et al., 2013). We think it can also be used as a signal of positive selection to identify mutations associated with genomic clusters. We reasoned that SMs with high clustering rate contributing to the enhanced transmissibility of lineage 2 should also be result of positive selection that is detectable as convergent or parallel evolution. SMs showed an unexpectedly high level of convergence among lineage 2.2.1, suggesting the action of selection.

From the above, it can be concluded that *ppsA* (3,249,025), *pks12* (2,302,033) and *pks13* (4,256,210) of lineage 2 were the final and meaningful mutation sites screened in our study, based on the results and analysis.

### 3.5 Sensitivity analysis

In the sensitivity analysis, the lineage 2 and lineage 4 data were divided into four groups and then reanalyzed using an ordinal regression analysis. As shown in Table 1, the first, second, third, and fourth group included non-clustered isolates, small clusters containing two isolates, clusters containing 3 to 6 isolates, and clusters containing ≥7 isolates, respectively. Only the SMs that were statistically significant in the univariate analysis and were risk factors were included in the statistical model.

As show in Table 7, *ppsA* (3,249,025), *pks12* (2,302,033), and *pks13* (4,256,210) of L2 were statistically significant and were risk factors in the ordinal regression analysis. Interestingly, the results for the ordinal and multivariate regression analysis were the same. The P and OR results for lineage 4 were undetermined, this can be attributed to the large standard error. The SM *ppsA*(3,249,025) was also more likely to be clustered than other SMs. Compared with non-clustered and small isolates, the larger and largest clustered isolates had higher clustering rate in the *ppsA* (3,249,025), *pks12* (2,302,033), and *pks13* (4,256,210) genes. The sensitivity analysis results did not change significantly compared to those of the univariate and multivariate regression analysis. The results of ordinal regression analysis based on the size of clustered isolates were like the main findings: SMs [*ppsA* (3,249,025), *pks12* (2,302,033), and *pks13* (4,256,210)] were risk factors for TB transmission.

**TABLE 7:** Deleterious effect of SMs on PKS proteins^a^.

Genomic position*	Nucleotide change	Amino acid change	Protein prediction	Protein domain*
*ppsA*				
3,249,025	T=>G	L1194R	Large decrease of stability	Linker*
3,248,074	GC=>AT	R877H	Large decrease of stability	Acyltransferase
3,247,865	GCAAA=>TAGGG	AQN807ARD	Large decrease of stability	Acyltransferase
3,247,851	GCCCGG=>ACTCGC	AR803TR	Large decrease of stability	Acyltransferase
*Pks8*				
1,885,385	T=>G	L1228V	Large decrease of stability	Linker*
*Pks12*				
2,302,033	G=>A	R1652H	Large decrease of stability	Enoyl reductase 1
Pks13				
4,256,210	G=>T	A1646S	Large decrease of stability	Linker*

^a^ Functional impact of the SMs on protein structure and function was predicted on one protein prediction algorithms, I-Mutant v2.0 (http://folding.biofold.org/i-mutant/i-mutant2.0.html). *Linker is the noncatalytic protein domain that connects different functional proteins. *Protein domain where the mutation occurs was checked in Uniprot database according to the protein sequence.

### 3.6 Deleterious effect of SMs on proteins

The SMs were predicted to negatively affect the respective proteins that affect the protein instability in nearby structural areas (Table 7). We also checked the Uniprot database for the protein domain where the mutation occurs according to the protein sequence (Trivedi et al., 2005; Siméone et al., 2010). P*psA* (3,249,025) and *pks13* (4,256,210) occurs in linker, while *pks12* (2,302,033) occurs in active site. Linker was found to be the noncatalytic protein domain that connects different functional proteins.

## 4 Discussion

Genetic diversity analysis revealed that the majority of these isolates belonged to lineage 2(the predominant sublineage was 2.2.1), with lineage 4 accounting for a significant proportion, while lineage 3 and lineage 1were less frequent. In addition, lineage 2 exhibited a higher clustering rate compared to lineage 4. These findings suggest that Beijing strains were more geographically dispersed compared to lineage 4, which are consistent with previous research (van Soolingen et al., 1995; Pang Y et al., 2012; Liu et al., 2018b). The overwhelming majority of TB cases in China were caused by L2 and L4 strains. The result of analysis also reminds us of the need to prioritize resources in cases where contact tracing is most likely to yield results. In China, it may be beneficial to direct contact tracing resources to lineage 2 and lineage 4 cases, as they pose the greatest risk of onward transmission resulting in new active TB cases.

We identified three SMs of lineage 2 in the *ppsA* (3,249,025), *pks12* (2,302,033), and *pks13* (4,256,210) gene regions that can potentially improve TB transmission. These SMs were predicted to alter the function of their respective proteins, supporting the hypothesis that they may affect TB transmission. Several biological and biochemical studies have determined the importance of the identified genes, which have proved critical to the virulence of *Mtb* in several animal studies (Kondo E Fau - Kanai and Kanai, 1972; Kolattukudy et al., 1997; Glickman and Jacobs, 2001; Sirakova et al., 2003). Furthermore, the results of this study are supported by previous genomic epidemiological articles (Onwueme et al., 2005; Trivedi et al., 2005; Gokhale et al., 2007b; Chopra et al., 2008; Quadri, 2014).

The *ppsA* gene is one of the clusters of *ppsABCDE* genes that has been shown to be involved in the biosynthesis of phthiocerol products (Figure 1A). The biosynthesis of phthiocerol products requires almost 24 catalytic activities on five large multifunctional modular proteins (Trivedi et al., 2005). Thus, if there is a mutation in one of the *pps* genes that can change protein function, it may increase or decrease the efficiency of this specificity of hand-to-hand transfer of the chain from one pps protein to another. The pks12 protein is involved in biosynthesis of a phospholipid MPM (Matsunaga et al., 2004). A study by Sirakova et al. (2003) showed that the growth and virulence of mutant pks12 was attenuated in an *in vivo* murine model (Sirakova et al., 2003). In mycolic acid synthesis, ps13 performs Claisen condensation of a C26 α-alkyl branch and C40–60 meromycolate precursors as the final assembly stage (Portevin et al., 2004). According to Alland et al. (2000), there is a novel class of thiophenes that prevent fatty acyl-AMP loading on pks13, interfere with mycolic acid biosynthesis, and have bactericidal effects on *Mtb* (Alland et al., 2000; Wilson et al., 2013). Aggarwal et al. (2017) found a novel benzofuran class lead molecule that targets *pks13* with fantastic drug-like characteristics and excellent pharmacokinetic and safety features that are active against MDR and XDR *Mtb* clinical strains *in vitro*.

In addition, we predicted the impact of SMs on protein structure. Mutations in *ppsA*, *pks12*, and *pks13* genes affect instability in nearby structural areas, which may affect nearby biological functions. Modular PKSs are multidomain proteins. Each module contains at least three essential domains, which are catalytic sites or active sites, namely, acyl transferase (AT), acyl carrier protein (ACP), and keto synthase (KS) domains. These catalytic sites or active sites are interconnected by small stretches of relatively unconserved sequences called linkers, which are more than covalent connectors (Gokhale and Khosla, 2000). Some SMs occur on active sites while others occur on linkers. Apparently, if the mutation occurs at active sites, it can affect the function of the *pks* gene. New progress has shown that linkers play a strong role in building the structural and functional assemblies of these diverse modular proteins in signal transduction and polyketide biosynthesis (Briggs and Smithgall, 1999; Gokhale et al., 1999; Xu et al., 1999; Gokhale and Khosla, 2000). Chopra et al. (2008) found that these linkers play an important role in the formation of docking domains through interacting helices. This study also showed that single amino acid substitutions in the linkers had an effect on the catalytic rates of product formation (Chopra et al., 2008). Similar studies based on the erythromycin PKS have shown the crucial role of single amino acids in forming a docking complex (Weissman, 2006). Thus, if the mutation occurs in linkers, it can also have an impact on protein-protein interactions and affect catalysis (Chopra et al., 2008). Since the positions of the modules can be changed by suitable linker engineering (Gokhale et al., 1999), it is worth studying the mechanism of linker action in chemical biology.

In conclusion, this study presents evidence through statistical analysis that three *Mtb PKS* genes in lineage 2 may contribute to disease progression and higher transmission of certain strains. Previous studies suggest that virulence change is caused not by mass nonsynonymous mutations, but rather by several critical mutations that affect gene product activity (Hershberg et al., 2008; Mikheecheva et al., 2017). Distinct lipids in the cell wall of mycobacteria synthesized by the three genes are critical to the pathogen’s ability to survive in the host’s hostile environment. Their production involves a complex process that requires many enzymes (Mehra et al., 1984; Chan et al., 1989; Vachula et al., 1989). When these lipids are lost due to mutation, *M. tuberculosis* becomes less virulent in the host (Camacho et al., 1999; Cox et al., 1999). This process offers multiple ways to intervene in lipids production and thus opens up many possibilities for designing antimycobacterial agents. It might be possible to view the three SMs as specific targets for the development of medications for the treatment of mycobacteria-related infections in people. Notably, the OR of *ppsA* (3,249,025) in lineage 2 were larger and the mutation was more likely to be clustered compared to other SMs. Perhaps we should pay more attention to SNP: ppsA (3,249,025) in the following study. The SNP [ppsA (3,249,025)] should be further evaluated with animal and immunological experiments to test its importance regarding biological impact and as a new drug target.

## 5 Strength and limitations

This study has several limitations. First, we did not conduct animal and immunological experiments to find biological support for the SMs identified in this study. Second, we lack key host factors that may influence disease transmissibility, such as age, host immune status, and pulmonary cavitation, to rule out the effect of confounding factors, which could reveal independent effects of SMs influencing transmissibility. Finally, for the small sample size of lineage 4, hidden mutation sites may not be screened out. We cannot tell if the SMs of lineage 4 and lineage 2 were the same or different. Of course, the sample size of lineage 2 is large enough. The SMs we found were more reliable, which could provide credible data for TB prevention and treatment.

## Data Availability

The newly sequenced whole genome dataset of 1,449 *M. tuberculosis* strains has been submitted to the NCBI (https://www.ncbi.nlm.nih.gov/) under the accession number PRJNA1002108. 1755 other isolates were acquired from nine previously published articles (Supplementary Table S1). Additional data can be obtained upon request by contacting the corresponding authors.
