# Supplementary material for: Polyketide synthases mutation in tuberculosis transmission revealed by whole genomic sequence, China, 2011–2019
Source: Front Genet. 2024 Jan 8;14:1217255. doi: 10.3389/fgene.2023.1217255 (PMC10800454; doi:10.3389/fgene.2023.1217255)
Supplement: Supplementary file 1 [file Table2.docx]

| Basic information | | | | Univariate regression analysis | | Multivariable regression analysis | |
| --- | --- | --- | --- | --- | --- | --- | --- |
| Genomic position | No. of mutationisolates | No. of clustered mutation isolates | Clustering rate | OR (95%CI) | P | OR (95%CI) | P |
| *PpsA* | | | | | | | |
| 3248074 | 1920 | 1057 | 55.05% | 1.23(1.02,1.48) | **0.026** | .807（0.593，1.100） | 0.175 |
| 3247851 | 2176 | 1217 | 55.93% | 1.66(1.33,2.07) | **<0.001** | 0.830(0.333,2.066） | 0.688 |
| 3247865 | 2160 | 1210 | 56.02% | 1.67(1.35,2.08) | **<0.001** | 1.305(0.499,3.415） | 0.588 |
| 3249025 | 2590 | 1399 | 54.02% | # | 0.96 | # | # |
| *Pks12* | | | | | | | |
| 2302033 | 2102 | 1187 | 56.47% | 1.72(1.40,2.11) | **<0.001** | 2.228(1.408,3.525） | **0.001** |
| *Pks13* | | | | | | | |
| 4256210 | 2582 | 1397 | 54.11% | 3.53(0.81,24.2) | 0.12 | # | # |
| *Pks8* | | | | | | | |
| 1885385 | 2585 | 1394 | 53.93% | * | 0.96 | # | # |

Univariate and Multivariable regression analysis on SMs associated with clustering in PKS gene region of lineage 2.2.1

#means there is no result in statistical software or the result was too large and nonsense. OR, odds ratio.

In terms of SNPs, isolates that possess the SNP in the PKS gene region are referred to as mutation isolates. The bold values mean these mutations were statistically significant.
