# Supplementary material for: Polyketide synthases mutation in tuberculosis transmission revealed by whole genomic sequence, China, 2011–2019
Source: Front Genet. 2024 Jan 8;14:1217255. doi: 10.3389/fgene.2023.1217255 (PMC10800454; doi:10.3389/fgene.2023.1217255)
Supplement: Supplementary file 3 [file DataSheet1.docx]

######Univariate and multivariate logistic regression analysis

library(openxlsx)

data <- read.xlsx("pks位点L2.xlsx", sheet = 1)

head(data)

rownames(data) <- data$sample

data <- data[,-1]

data$cluster <- factor(data$cluster)

################################################

head(data)

library(dplyr)

res_GLM <- glm(cluster~a1877744, data=data, family = binomial()) %>% summary()

data.frame(OR = exp(res_GLM$coefficients[2,"Estimate"]),

CImin = exp(res_GLM$coefficients[2,"Estimate"]-1.96*res_GLM$coefficients[2,"Std. Error"]),

CImax = exp(res_GLM$coefficients[2,"Estimate"]+1.96*res_GLM$coefficients[2,"Std. Error"]),

P_value = res_GLM$coefficients[2,4])

############Ordered logistic regression analysis code:

library(MASS)

library(tidyverse)

##############################

df1 <- df %>%

dplyr::mutate(

across(edu, ~ fct_inseq(., ordered = TRUE)) # y, 有序分类变量设置为order

)

fit1 <- polr(edu ~ sex + urban,

data = df1,

method = c("logistic")

)

summary(fit1)
